# Exploring the complexities and challenges of healthcare access for transgender people in Minas Gerais state: a qualitative study a decade following the implementation of the transsexualization process in the Brazilian National Health System

**DOI:** 10.1590/S2237-96222024v33e2024350.especial.en

**Published:** 2024-12-13

**Authors:** Samuel Araujo Gomes da Silva, Paula Miranda-Ribeiro, Kenya Valeria Micaela de Souza Noronha, Gilvan Ramalho Guedes

**Affiliations:** 1 Universidade Federal de Minas Gerais, Faculdade de Medicina, Belo Horizonte, MG, Brazil; 2 Universidade Federal de Minas Gerais, CEDEPLAR, Belo Horizonte, MG, Brazil

**Keywords:** Salud, Personas Transgénero, Minorías Sexuales y de Género, Equidad en el Acceso a los Servicios de Salud, Health, Transgender People, Sexual and Gender Minorities, Equity in Access to Health Services

## Abstract

**Objective:**

This article explores the complexities and challenges of healthcare access for transgender people in Minas Gerais.

**Methods:**

**:** This study is based on a subsample from the *Manas Survey*, comprised of 15 semi-structured interviews with transgender people, conducted between May 2018 and May 2020, analyzed using the content analysis method and organized into a thematic network.

**Results:**

**:** The results highlight the need for adequate structures, specific training for healthcare professionals, the influence of support networks and challenges in mental health.

**Conclusions:**

**:** Despite advances in transgender health policies, access barriers persist, underscoring the importance of training strategies, accurate record-keeping and the expansion of specialized services and the role of primary health care. The study makes progress by including the perceptions from residents of small and medium-sized cities across different regions of the state, aiming to improve access and care for the transgender population via the Brazilian National Health System in Minas Gerais.

## Study contributions

**Main results:**

The findings underscore the need for adequate structures, specific training for healthcare professionals, the influence of support networks and challenges in mental health, in addition to including the perceptions of residents from small and medium-sized cities.

**Implications for services:**

Improving the use of social name and gender identity in records. There is a need for training healthcare professionals in gender identity, expanding transgender-specific services and the role of primary health care (PHC) in this topic.

**Perspectives:**

Expansion of specialized services and the role of PHC for this population, in order to improve access and care for the transgender population via the Brazilian National Health System in Minas Gerais state.

## RESUMEN

**Objetivo:**

Este artículo explora las complejidades y desafíos del acceso de personas trans a los servicios de salud en Minas Gerais.

**Métodos:**

Se basa en una submuestra de la “Pesquisa Manas”, compuesta por 15 entrevistas semiestructuradas a personas trans, realizadas entre mayo de 2018 a mayo de 2020, examinadas mediante la técnica de análisis de contenido y organizadas en red temática.

**Resultados:**

Los resultados muestran la necesidad de estructuras adecuadas, capacitación de profesionales, influencia de redes de apoyo y desafíos en salud mental.

**Conclusión:**

Pese a los avances en las políticas de salud trans, las barreras de acceso se mantienen, se resalta la importancia de capacitación, registros precisos y expansión de los servicios especializados y de atención primaria. El estudio incluye las percepciones de los residentes de ciudades pequeñas y medianas, con vistas a mejorar el acceso y atención a la población trans dentro del Sistema de Salud Único en Minas Gerais.

## INTRODUCTION

The 1988 Federal Constitution established health as a universal and comprehensive right, reflecting the ideals of health reform. However, its implementation for specific groups, such as the transgender population, remains a distant goal. The discrepancy between the constitutional ideal and the real experience of these individuals in healthcare services results from the lack of effective mechanisms that facilitate their access to the Brazilian National Health System (*Sistema Único de Saúde* - SUS).^1^

The first specific actions aimed at including transgender people in public policies formulated and implemented by the nation date back to 2004, leading to the adoption of concrete measures, such as the Transsexualization Process in the SUS since 2008,^2^ which was expanded in 2013 to include trans men and transvestites. The 2006 *SUS Users’ Rights Charter* also introduced the use of social names to integrate transgender people into health services.

The transsexualization process comprises a set of policies, actions and health services aimed at providing assistance and various procedures for transgender people, such as hormone therapy, gender-affirming surgeries and psychosocial support. Access to this process requires certain criteria, such as a minimum age of 18 to start hormone therapy and 21 years old for sex gender-affirming surgeries. The surgery requires a specific recommendation, after two years of follow-up by a multidisciplinary team. The implementation of these requirements faces criticism due to the centrality of the biomedical evaluation, the length of the process and the lack of respect for the use of social names, perpetuating gender stereotypes and limiting the autonomous construction of identity narratives, thereby restricting the validity of the transgender condition to the team’s diagnosis.^3,4^

In addition to the transsexualization process, other essential and comprehensive needs of transgender individuals that could contribute to the promotion of comprehensive health are often neglected. This discussion highlights a dilemma related to the removal of transsexualism from the list of mental disorders in the ICD-11, redefining it as gender incongruence. While some support depathologization in order to reduce medicalization and dependence on reports to validate transsexual identities, others are concerned about the possible loss of trans - specific services.^3-4^

Some of these concerns could be addressed by the National LGBT Comprehensive Health Policy (*Política Nacional de Saúde Integral LGBT*), established in 2013, and by the State LGBT Comprehensive Health Policy of Minas Gerais,^5^ instituted in 2020. These policies aim to legitimize specific demands of this group, guide comprehensive care, and combat discrimination in health services.^6^ However, barriers persist due to the lack of adequate training of health professionals, resulting in cisheteronormative attitudes.^7-9^ These attitudes drive transgender people away from health services, often leading them to seek care only when it can no longer be postponed.^10^

Although the transgender movement has made remarkable progress, translating these achievements into effective public policies remains a significant challenge in Brazil. Expanding access to specific and comprehensive health services for transgender people and transvestite is crucial to ensuring full recognition of their citizenship and overcoming the violence faced by this community. ^11^ In order to understand the obstacles to expanding health policies for the transgender population, this article aims to explore the complexities and challenges of healthcare access for transgender people in Minas Gerais state.

## METHODS

In order to address the proposed objective, this article uses part of the Manas Survey (*Pesquisa Manas* - PMa), which assessed health-related quality of life and the adequacy of the health system in meeting the needs of the LGBT+ population in Minas Gerais. The PMa is a mixed-method study (quantitative- qualitative) comprising four phases. In phase 1, semi-structured interviews were conducted with specialized healthcare providers and public policy managers for the LGBT+ population in the state of Minas Gerais. Phase 2 involved online interviews with a sample of lesbians, gays, and bisexuals from all regions of the state of Minas Gerais. Phase 3 consisted of semi-structured interviews with people who had also participated in phase 2. Finally, phase 4 comprised semi-structured interviews with transgender people. Data collection occurred between May 20, 2018 and May 20, 2020. Since the study involved primary data collection, it was submitted for ethical appraisal to the National Research Ethics Committee (CAAE – 85561717.0.0000.5149), and was approved on May 15, 2018. All participants had access to the Free and Informed Consent Form and agreed to participate.

For this specific study, the analysis uses a qualitative dataset consisting of semi-structured interviews with transgender people aged 18 years or older and living in ten municipalities in Minas Gerais, which constitute phase 4 of the PMa. Fifteen individuals residing in the municipalities that are home to seven of the 13 health macro-regions of the state were interviewed. The interviews were conducted according to the availability of respondents and interviewers.

The interviews were conducted using a script based on the literature on the topics covered in the study (self-perception of health, experience of using healthcare services, experiences of not using healthcare services and mental health).

Data was organized and investigated using the content analysis (CA) method.^12^ This data processing technique aims to interpret the respondents’ statements through a description aligned with the predefined and/or emerging categories of analysis during the process. In addition, it allows for an in-depth and diverse reading of the data collected,^13^ which facilitates the understanding of communication elements, such as “manifest or latent content and explicit or hidden meanings”.^14^

In order to enhance the understanding of the interview results, we combined the structure of content analysis with thematic networks, a qualitative analysis tool used to organize and represent themes emerging from textual data, structuring them into three levels: basic codes, organizing themes, and global themes.^15^ This analytical approach is characterized by the organization of data into a network of meanings, taking into account six main steps: coding the material; identifying themes; constructing the thematic network; describing and exploring it; creating a summary structure of patterns and connections within these networks; and analyzing it according to the theoretical assumptions of the research.

In order to begin the analysis process, all interviews were transcribed verbatim and uploaded into the NVivo software version 12 pro to streamline, enhance possibilities and ensure reliability in the analysis.^13^ The categories for coding the material were predefined based on the literature collected and, once the interview content was organized within each category, the comparative visualization tools of the categorizations were used to assist in identifying themes and constructing the thematic network.

## RESULTS

According to Table 1, the 15 people interviewed are concentrated in the younger age groups. Regarding gender identity, seven people identified as female, six as male and two as agender/non-binary. With regard to sexual orientation, eight people reported being heterosexual, four bisexual/pansexual, one lesbian, one gay and one demisexual.

**Table 1:** Composition of transgender people interviewed, the Manas Survey, Minas Gerais state, Brazil, 2018-2020

Identification	Age group	Gender identity	Sexual orientation
T01	18-24	Woman/female	Demisexual
T02	40+	Woman/female	Heterosexual
T03	25-29	Woman/female	Heterosexual
T04	25-29	Man/male	Heterosexual
T05	25-29	Woman/female	Heterosexual
T06	25-29	Man/male	Bisexual/pansexual
T07	18-24	Man/male	Heterosexual
T08	18-24	Agender	Bisexual/pansexual
T09	40+	Woman/female	Heterosexual
T10	40+	Woman/female	Lesbian
T11	25-29	Man/male	Gay
T12	18-24	Non-binary	Bisexual/pansexual
T13	30-34	Man/male	Heterosexual
T14	35-39	Man/male	Heterosexual
T15	18-24	Transvestite	Bisexual/pansexual

**Figure 1:**
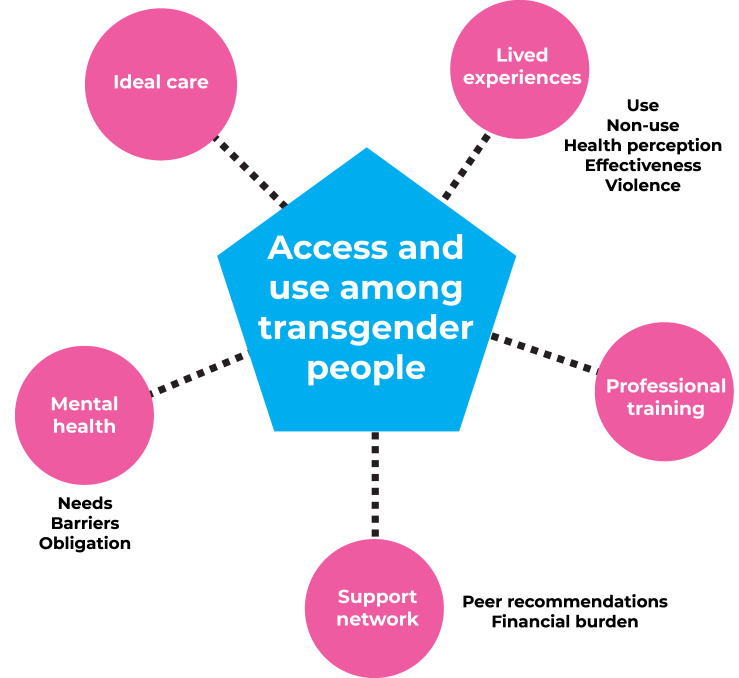
Thematic network, focusing on access to and use of health services by transgender people, Manas Survey, Minas Gerais state, Brazil, 2018-2020

The analysis of the interviews resulted in a thematic network comprised of five themes (Figure 1). The first, called *lived experience*, addresses how the life experiences of these people were shaped and shaped their patterns of use and non-use of health services, their perception of health status, the effectiveness of care received and, mainly, the physical and symbolic violence suffered in these spaces. Subsequently, we present the second theme, with reports on how the *training of health professionals* (or lack of training) affects the willingness of transgender people to seek health services and the care they receive. The third theme, *support networks*, addresses how peer recommendations influence the choice of healthcare professionals and procedures, with or without medical supervision and with or without the financial means available to pay for the desired procedures. The fourth theme addresses challenges related to *mental health*, especially the mandatory monitoring for the transsexualization process and its consequences, as well as the barriers to accessing such services. Finally, the fifth theme deals with the respondents’ perception of what constitutes *ideal care* for transgender people in health services.

The *lived experiences* of the transgender population in health services are the result of a series of social and contextual elements, which shape not only their perceptions of “being healthy”, but essentially the relationship between their desires and ideals of self-image and what is offered in health services. When asked about their self-rated health compared to other transgender individuals in the same age group, the impact of common experiences in transgender people’s lives on health outcomes becomes more evident. Experiences such as using hormones without medical supervision, injecting industrial silicone, excessive drug use, exposure to various risks associated with prostitution, the centrality of body modifications in the construction of self-esteem and the impacts of the cumulative effects of all these factors throughout life are some of the points cited as reasons for their health classification. (Table 1, box a).

It was also mentioned that, often, seeking health services was not even performed, leading to the normalization of practices that are widely recognized as inadvisable – such as self-medication and other procedures without supervision of a health professional – among these individuals. However, self-perception of health was often related to the availability of care. For people who manage to maintain regular follow-up in health services combined with self-care practices, there seems to be an expansion of the understanding of what it means to have good health, associating it with consistent use of these services (Table 1, box b).

Most of the health demands reported are related to the transition processes – hormone therapy and surgeries. A significant portion of these processes is linked to the development of self-esteem regarding one’s own body and gender identity. Nevertheless, even with the establishment of specialized services for transgender people, they remain limited to large urban centers and face challenges in providing services and supplies that meet the demands of this population on a regional basis. In order to receive specialized care, many individuals have to travel from their municipalities and even from their states. The transsexualization process is restricted to a few services, time-consuming and has long waiting lists, which can extend up ten years for surgical procedures. It is worth highlighting that surgery is not a necessity for all transgender people. In some reports, it is possible to identify experiences in which gender affirmation surgery is neither desired nor central to the development of their identities (Table 1, box c).

Regarding the experience in healthcare facilities, it is acknowledged that the challenges faced by the general population, derived from other social characteristics, such as income, race/skin color and schooling, intersect with prejudice against transgender individuals. For some of the interviewees, the fact of being a transgender person makes the experience of using services worse. However, gender identities were not generally the reason for poor service; rather, it was the result of seeking emergency services and finding overcrowded facilities with limited availability of professionals for adequate care. However, the use of emergency services, in the majority of cases, may be the result of a delay in seeking care in primary health care (PHC) services, motivated by the fear of being poorly cared due to their gender identity (Table 1, box d).

The frequency of use of health services is also heterogeneous. When asked about the last time they visited a health service, some people reported having had few experiences, a long time ago, while others described themselves as frequent users of health services with recent prior experiences. On the other hand, the effectiveness of care is perceived positively when the follow-up takes into account both the demands of hormone therapy and the general physical and mental health conditions (Table 1, box e).

The likelihood of transgender people seeking care encompasses both objective issues (e.g., disrespect for the social name in services for those who have not legally changed their names and the lack of recognized safe and welcoming spaces for transgender people outside major urban centers) and social issues, which, combined with the quality of care, create a sense that seeking care is a waste of time. Similarly, negative experiences within the healthcare system are permeated by rights violations both inside and outside the medical offices. Among the reports, some mentioned the need to seek specialized care outside the municipality of residence, without any support from the SUS, resulting in costs exceeding their available income; disrespect for their social name, including in trans-specific equipment; unethical and disrespectful conduct by healthcare professionals during care, such as refusal to prescribe hormones and perform a masculinizing mastectomy due to personal disagreement with the removal of a healthy breast (Table 1, box f).

Despite the predominance of negative reports regarding the care provided to transgender people, some positive experiences were also mentioned, with emphasis on the role of healthcare professionals in welcoming and caring for transgender people. The willingness to seek the best possible treatment for patients’ needs, the demonstration of mastery of the procedures and techniques to be applied, and especially the humane and dignified treatment made a difference, according to the reports of transgender people. For some of them, factors such as being a well-known person and/or paying for the service may have facilitated the process (Table 1, box g).

*The training of healthcare professionals* is cited as a factor of utmost relevance to the experience of transgender people in health services. For many interviewees, professionals currently working in health systems lack the technical qualifications necessary to address with the needs of the LGBT+ population adequately. In most cases, these professionals do not have the training required to properly care for this population. Those who have received training are often the only ones addressing such needs within the service or in the municipality, and when they propose discussions about the demands of the transgender population among their colleagues, they face resistance (Table 2, box a).

**Box 1:** Selected statements on the lived experiences of transgender people in health services, Manas Survey, Minas Gerais state, Brazil, 2018-2020

**a**	“I think [my health] is good, I’m not out freezing on the street at night [places of prostitution], feeling cold all night, being out there in the early hours in skimpy clothes [...] I’ve seen cases of trans women in my age group who had their legs completely damaged because of industrial silicone.” (*Trans woman, White, 40 years or older)*
	“[her trans friends] self-administered hormones, they don’t have access to a gynecologist. Those who need medication can get it on the black market [sic], they control their hormone levels on their own and this is a ticking time bomb that affects their psychological state, their emotions, everything.” (*Trans man, mixed-race, 30-34 years old*)
**b**	“I think that despite having self-administered hormones for so long, I had a *check-up a year ago*, everything was fine, it could have gone wrong, especially since I used to take two ampoules a week and, now I take them every 15 days, it’s totally different, but yes, I’m in good health and I have access.” (*Trans man, Black, 35-39 years old*)
	“My tests are all good. I’m on the right medication, right?! It isn’t Perlutan, which is a contraceptive, and is terribly harmful, even for cis women.” (*Transvestite, Black, 18-24 years old*)
**c**	“I don’t need a general practitioner because I don’t feel anything, my health is great. I need endocrine and psychological care, as a transgender person.” (*Transvestite, Black, 18-24 years old*)
	“[...] at the outpatient clinic, that’s basically it, just psychological and psychiatric follow-up, because we don’t have the medication available via the SUS. So, for those who don’t work, they can’t afford to transition via the SUS.” (*Trans woman, White, 25-29 years old*)
	“The only place that is close to us is in BH, which is a hospital for monitoring transsexuality and such, psychological support, hormones, for this region there is nothing here, there is nothing.” (*Trans man, mixed-race, 34-34 years old*)
**d**	“The treatment was good, it could have been better if there had been a doctor [at the UPA], they were in the middle of a shift change [...], but I didn’t feel excluded because it was for everyone, I was there just like everyone else.” (*Trans woman, White, 25-29 years old*)
	“[...] there isn’t adequate [care], not because I’m trans. It’s because the care is really poor, very superficial, health care in general is bad, even for people who aren’t transgender. Doctors don’t even look at you, there’s a lot of that, doctors don’t even look, you know, I think it’s a widespread problem and I think trans people will suffer a lot of prejudice, but I will probably suffer when you need it most.” (*Trans woman, mixwd-race, 40 years or older*)
**e**	“[...] I haven’t known what a gynecological exam is for years. Pap smear, which is something that every person with female reproductive system should have, I haven’t done.” (*Trans man, mixed-race, 34-34 years old*)
	“[...] I used the health service last year, the PSF. I don’t go often, but once or twice a year.” (*Trans woman, White, 40 years or older*)
	“I noticed [a difference] completely. I was on a cycle [of hormone therapy], and she [the doctor] said we’re not going to continue with your cycle. She ordered a blood test and saw that it wasn’t good, so she changed it. And there’s already been a lot of change. From December to now, there’s been a lot of change.” (*Trans man, White, 18-24 years old*)
**f**	“They [trans friends] are hesitant, right? They are not treated well. Those who don’t seek care is because their name has not been rectified yet. So, it’s more about fear, lack of respect from the healthcare professional.” (*Trans woman, White, 40 years or older*)
	“Let’s start with social class. I’ve always been poor, so accessing public health services has always been very slow and difficult for me. So, I never had this habit of having sore throat and going to the health center, or having a fever and going to the UPA. We go to the pharmacist because it’s faster, cheaper, easier, there is a routine, there is work, we can’t waste time with this kind of thing.” (*Trans man, mixed-race, 25-29 years old*)
	“[...] we were there and they came in and said, ‘oh no, the patients with gender dysphoria’ Everywhere you go, you always have a specific label, you don’t have your identity.” (*Trans woman, White, 25-29 years old*)
**g**	“[...] she [the polyclinic attendant] asked “why doesn’t she want to show her ID?” He [the gynecologist] said it wasn’t necessary and took me with him. He is still my gynecologist today, otherwise I wouldn’t have left there with hormones, but it was a more complicated time 24 years ago.” (*Trans woman, White, 40 years or older*)
	“[...] he [the doctor] was not invasive in trying to find an explanation for my condition, he treated me like a normal person, just as I am. [...] I didn’t feel violated, I was finally taken care of. The doctor ordered a [large] number of tests, everything came back fine, there were no changes in anything, it was super easy.” (*Trans man, mixed-race, 25-29 years old*)

The difficulties in finding suitable professionals to address specific health issues of transgender patients are due to this lack of education and training to care for this population. Thus, in order to find professionals or to self-medicate/undergo a procedure, this population group frequently relies on their *support networks*. Usually, this recommendation comes from a close friend who has already been seen by this professional and had a positive experience. In the absence of care, friends with similar experiences assist in prescribing over-the-counter medications or in applying industrial silicone (Table 2, box b).

**Box 2:** Selected statements on factors influencing the use of health services by transgender people, Manas Survey, Minas Gerais state, Brazil, 2018-2020

a) Professional training	“[...] I had to help the doctor who treated me. [Tell him] that my levels shouldn’t drop below that or I would menstruate. If I went above that, the testosterone would turn into progesterone and then I would have more female hormones. I had to sit down with the endocrinologist and tell him about it. So I’m still doing it on my own, you know.” (Trans man, mixed-race, 34-34 years old)
	“Because, generally, we have professionals who don’t understand, just like I told you. They don’t know what to call it. I’ve seen cases of girls who were prescribed medication by the doctor, while asking what they had been taking. And that’s where it ends.” (Transvestite, Black, 18-24 years old)
b) Support networks	“[...] I heard about him when I went to my psychiatry appointment, I exchanged ideas with some girls who had already undergone surgery, I did some research and he inspired confidence.” (Trans woman, White, 25-29 years old)
	“[I found him] through groups of trans people in Belo Horizonte. There, they always share a list of the best doctors in Belo Horizonte and São Paulo. This doctor I looked for is one of them, and he doesn’t even charge [for consultations]. He only charges trans people for surgery, he doesn’t charge when you go there, he doesn’t charge for the consultation.” (Trans man, Black, 35-39 years old)
	“[...] it was adequate, I emphasize again, [but] her consultation was very expensive. This doctor is considered the only one in [the municipality of residence]. The one who provides care for women, trans people in general. But she is very expensive, it can be very unfeasible to have a consultation, to have follow-up care with her.” (Trans woman, undeclared race, 18-24 years old)
	“[...] if I get a spot at the clinic, the hormone is free, but the problem is transportation. Even so, it costs the same as the capsule, around 90 reais for transport.” (Non-binary person, Black, 18-24 years old)
c) Mental health	“The psychologist there is awful. You don’t spend ten minutes with her in the room. I think it’s just bureaucracy. It’s like hi, hi, how are you? Bye, sign here for me.” (Trans man, White, 18-24 years old)
	“I’ve had terrible experiences with psychologists, you know?! The kind of psychologist who really pathologizes , you know?! They don’t understand. And they treat my existence as a universal issue, outside of my social context, gender context, gender identity, and everything else. Which was terrible!” (Transvestite, Black, 18-24 years old)
	“[The psychologist] helped me a lot to soften some issues that I was dealing with and some stereotypical aspects of the female gender. She helped me to problematize in a way, not in a normative way, on the contrary, but in a really sensitive way.” (Trans woman, white, 25-29 years old)
	“[...] when you transition , it’s not just your exterior that changes, it has an impact on your social status. You leave one place and go to another. Before, I was seen as a woman, but nowadays, for example, I go out on the street and I’m not afraid to walk alone because I’m in a male space. All these things that affect you, you have to understand. And understanding this on your own is sometimes not very easy.” (Trans man, mixed-race, 25-29 years old)
d) Ideal care	“[...] that you receive attention in a minimum amount of time, you know? [...] a service where you can have a conversation and there is no shortage of supplies to assist you.” (Agender person, White, 18-24 years old)
	“I think the ideal is to understand their singularities, but without making them exotic. [...] to understand that it is not a pathological condition, that people will not be confused. [...] to consider the entire social, historical and political context. That in fact the problem is not just with the person and the suffering is not only theirs. They aren’t suffering because they’re not accepted. They’re suffering because there’s in fact a whole context that generates this suffering. The ideal system has to take into account these mental health issues and how society actually makes people sick.” (Trans woman, White, 25-29 years old)
	“[...] not mixing particular principles with care, because in health care you meet people of all types, from all classes, with various principles.” (Trans man, mixed-race, 34-34 years old)
	“A service that does not respect you in your entirety as a human being.” (Trans man, Black, 35-39 years old)

Although the support network assist in identifying professionals and procedures to meet healthcare needs, the cost is not always affordable. In many cases, people seek financial resources from relatives, purchase services in installments, or struggle to incorporate this cost into their budget, increasing the risk of impoverishment. Furthermore, even when treatments are free, they are not always offered in the municipality of residence, and the cost of transportation and accommodation can make them unfeasible in some situations (Table 2, box b).

Regarding *mental health*, for some interviewees, the mandatory psychiatric and psychological follow-up in order to undergo gender affirmation surgeries may serve as a common access to the system. On the other hand, this requirement may, in some cases, lead to procedural care, that is, protocol-based care, distancing this population from mental health services (Table 2, box c).

Despite questioning many of the processes involving compulsory psychological follow-up, many interviewees recognized the importance and role of this follow-up in the well-being of transgender people. For some people, when well-conducted, the follow-up helps not only with the transition itself, but also in dealing with the demands of the new social places where transgender people begin to live, as well as with the contextual changes that impact their lived experiences before and after the surgeries (Table 2, box c).

Reports on what constitutes *ideal care* for this population point to the need for humanized care that takes into account the specificities of transgender people, without trying to fit them into an explanation for their condition or a treatment to “solve the problem” (Table 2, box d).

In general terms, adequate care is understood as one where genuine attention is paid to the person’s problem, with sufficient resources and time for meaningful interaction. It is also essential that care is impartial and that the patient is treated with respect and dignity, considering their life history and lifestyle, not just their symptoms. The identity of each person must be respected, regardless of what is stated in official documents, and personal principles should be separated from professional duties. These are fundamental attitudes for care that is understood as inclusive and respectful.

## DISCUSSION

This study aimed to evaluate access to health services from the perspective of transgender individuals. Our findings align with other studies that highlight the main factors that affect whether or not people seek for healthcare. The key factors include the need for adequate structures to address demands, including the use of the social name,^16-18^ the recognition of specific needs though accurate diagnosis,^19^ the lack of training of professionals,^17,18^ the centrality of support networks in finding professionals,^18^ the high prevalence of self-medication and body changes without medical supervision,^20^ the sometimes compulsory and inadequate contact with mental health professionals,^19^ the limited number and regionally concentrated availability of services addressing the transsexualization process^2^ and the need to implement a comprehensive care approach that is not limited to the transsexualization process.^18^

The results show the need to expand training strategies for healthcare professionals, both in-service and during their education, within the framework of trans-specific health policies, especially in smaller municipalities. The adoption of these strategies would enhance knowledge and effectiveness of the health demands of this population. The perception of effective health care would increase the demand for services, including PHC services, which would lead to greater control and prevention of illness causes and risk factors in this population. Adequate follow-up of transgender patients would also have economic effects, both from the individual perspective and for the health system, by reducing the need for more complex, costly and long-term treatments.

The discussions presented have broad implications for transgender health policies, highlighting the need for improvements in information records to allow the use of social names and the inclusion of gender identity as a variable in medical records and registries, such as e-SUS. These measures would strengthen health surveillance, providing a better understanding of the epidemiological profile of the transgender population. Additionally, there is a need to train healthcare professionals in gender identity and its relationship with the social determinants of health. With regard to care, it is crucial to fully recognize and enable state trans-specific services and to expand the role of PHC in identifying and monitoring the transgender population. This would ensure an accurate understanding of the demand and the capacity of PHC in prescribing and monitoring hormone therapy for this population in primary healthcare centers. This recommendation is supported by the Brazilian Society of Family and Community Medicine,^21^ which points out that hormone therapy can be performed in primary health care with the support of secondary and tertiary level professionals, when necessary, thereby reducing referrals to specialized centers and facilitating access to services. This approach is essential, especially in Minas Gerais, given the uneven geographic distribution of specialized services in the state. In addition, it is imperative to expand mental health services, prioritizing respect for gender identity and addressing the life-long impacts of stigma and prejudice.^22^

This research has two significant limitations. The sample size and its non-representative nature highlight the need for more comprehensive future studies. Another limitation lies in the temporal context , given that perceptions and challenges may evolve over time. Further research should address these issues, exploring regional disparities and incorporating health professionals’ perspectives to develop effective strategies for improving access to the SUS for transgender people. Despite these limitations, the findings provide valuable insight into access to healthcare for the transgender population in Minas Gerais state. The analysis contributes to broadening the debate, as it considers a population that is understudied, especially among residents of small and medium-sized cities in various regions of Minas Gerais. As these locations are far from large urban centers, they present access difficulties that may be exacerbated by the nature of the health care required to meet the needs of this population group.
