# Supplementary material for: Exploring the complexities and challenges of healthcare access for transgender people in Minas Gerais state: a qualitative study a decade following the implementation of the transsexualization process in the Brazilian National Health System
Source: Epidemiol Serv Saude. 2024 Dec 13;33(spe1):e2024350. doi: 10.1590/S2237-96222024v33e2024350.especial.en (PMC11654818; doi:10.1590/S2237-96222024v33e2024350.especial.en)
Supplement: Translation

# Translation: Explorando as complexidades e os desafios do acesso à saúde para pessoas trans em Minas Gerais: um estudo qualitativo após uma década da implementação do processo transexualizador no Sistema Único de Saúde

**DOI:** 10.1590/S2237-96222024v33e2024350.especial.pt

**Authors:** Samuel Araujo Gomes da Silva, Paula Miranda-Ribeiro, Kenya Valeria Micaela de Souza Noronha, Gilvan Ramalho Guedes

**Affiliations:** 1 Universidade Federal de Minas Gerais, Faculdade de Medicina, Belo Horizonte, MG, Brasil; 2 Universidade Federal de Minas Gerais, CEDEPLAR, Belo Horizonte, MG, Brasil

**Keywords:** Saúde, Pessoas Transgênero, Minorias Sexuais e de Gênero, Equidade no Acesso aos Serviços de Saúde

## Abstract

**Objetivo:**

Explorar as complexidades e desafios do acesso de pessoas trans aos serviços de saúde em Minas Gerais.

**Métodos:**

Baseia-se em uma subamostra da *Pesquisa Manas*, composta por 15 entrevistas semiestruturadas com pessoas trans, realizadas entre maio de 2018 e maio de 2020, analisadas pela técnica de análise de conteúdo e organizadas em rede temática.

**Resultados:**

Os resultados revelam a necessidade de estruturas adequadas, formação específica para profissionais de saúde, influência das redes de suporte e desafios na saúde mental.

**Conclusões:**

Apesar dos avanços nas políticas de saúde trans, as barreiras de acesso persistem, destacando a importância de estratégias de treinamento, registros precisos e ampliação dos serviços especializados e da atuação da atenção primária à saúde. O estudo avança ao incluir as percepções de moradores de cidades de pequeno e médio porte de diferentes regiões do estado, visando melhorar o acesso e atendimento à população trans pelo Sistema Único de Saúde em Minas Gerais.

## Contribuições do estudo

**Principais resultados:**

Os resultados revelam a necessidade de estruturas adequadas, formação específica para os profissionais, influência das redes de suporte e desafios na saúde mental, além de incluírem as percepções de moradores de cidades de pequeno e médio porte.

**Implicações para os serviços:**

Melhoria do preenchimento de nome social e identidade de gênero nos registros. Necessidade de formação em identidades de gênero para profissionais de saúde, da expansão de serviços transespecíficos e da atuação da atenção primária à saúde (APS) no tema.

**Perspectivas:**

Ampliação dos serviços especializados e da atuação da APS para essa população, no intuito de melhorar o acesso e o atendimento à população trans pelo Sistema Único de Saúde em Minas Gerais.

## INTRODUÇÃO

A Constituição Federal de 1988 consagrou a saúde como direito universal e integral, refletindo os ideais da reforma sanitária, porém sua concretização para grupos específicos, como a população trans, permanece distante na prática. A discrepância entre o ideal constitucional e a experiência real desses indivíduos, nos serviços de saúde, resulta da falta de mecanismos eficazes que facilitem o acesso dessa população ao Sistema Único de Saúde (SUS).^1^

As primeiras ações específicas para a inclusão das pessoas trans em políticas públicas formuladas e executadas pelo Estado brasileiro são de 2004, o que propiciou a adoção de medidas concretas, como é o caso do Processo Transexualizador no SUS desde 2008,^2^ ampliado em 2013 para incluir homens trans e travestis. A *Carta de Direitos dos Usuários do SUS* de 2006 também introduziu o uso do nome social para integrar pessoas trans nos serviços de saúde.

O processo transexualizador se constitui em um conjunto de políticas, ações e serviços de saúde destinados a oferecer assistência e procedimentos variados para pessoas trans, como hormonioterapia, cirurgias de redesignação sexual e acompanhamento psicossocial. Para acessar o processo, são necessários certos critérios, como idade mínima de 18 anos para iniciar a terapia hormonal e 21 anos para cirurgias de redesignação sexual. A cirurgia requer uma indicação específica, após acompanhamento de dois anos por uma equipe multidisciplinar. A implementação desses requisitos enfrenta críticas, devido à centralidade da avaliação biomédica, à duração do processo e à falta de respeito ao uso do nome social, perpetuando estereótipos de gênero, o que limita a construção autônoma de narrativas identitárias e restringe a validade da condição transexual ao diagnóstico da equipe.^3,4^

Para além do processo transexualizador, há outras necessidades essenciais e abrangentes dos indivíduos trans que contribuiriam para a promoção de uma saúde integral, mas são frequentemente negligenciadas. Essa discussão aponta para um dilema relacionado à retirada da transexualidade do rol de doenças mentais no CID-11, classificando-a como incongruência de gênero. Enquanto alguns apoiam a despatologização, a fim de serem reduzidas a medicalização e a dependência de laudos para validar identidades transexuais, outros se preocupam com a possível perda de serviços transespecíficos.^3-4^

Parte dessas preocupações poderiam ser solucionadas pela Política Nacional de Saúde Integral LGBT, estabelecida em 2013, e pela Política Estadual de Saúde Integral LGBT de Minas Gerais,^5^ instituída em 2020. Essas políticas visam legitimar demandas específicas desse grupo, guiar o atendimento integral e combater a discriminação nos serviços de saúde.^6^ No entanto, as barreiras persistem, devido à falta de formação adequada dos profissionais de saúde, resultando em atitudes cis-heteronormativas.^7-9^ Essas atitudes afastam as pessoas trans dos serviços de saúde, as quais, na maior parte das vezes, irão buscar atendimento quando não há mais como postergar o cuidado.^10^

Embora o movimento trans tenha alcançado progressos notáveis, transformar essas conquistas em políticas públicas efetivas continua sendo um desafio substancial no Brasil. Ampliar o acesso a serviços de saúde específicos e abrangentes para pessoas trans e travestis é crucial para garantir o pleno reconhecimento de sua cidadania e superar a violência enfrentada por essa comunidade.^11^ No intuito de compreender os obstáculos na ampliação das políticas de saúde para a população trans, este artigo tem como objetivo explorar as complexidades e desafios do acesso de pessoas trans aos serviços de saúde em Minas Gerais.

## MÉTODOS

Para responder ao objetivo proposto, o artigo utiliza uma parte da Pesquisa Manas (PMa), que avaliou a qualidade de vida relacionada à saúde e a adequabilidade do sistema de saúde para lidar com as necessidades da população LGBT+ em Minas Gerais. A PMa é um estudo de método misto (qualitativo e quantitativo) composto por quatro fases. Na fase 1, foram conduzidas entrevistas semiestruturadas com prestadores de serviços de saúde especializados e gestores de políticas públicas para a população LGBT+ no estado de Minas Gerais. Na fase 2, foram realizadas entrevistas *online* em uma amostra de lésbicas, gays e bissexuais de todas as regiões do estado de Minas Gerais. A fase 3 consistiu em entrevistas semiestruturadas com pessoas que também tinham respondido à fase 2. Por fim, a fase 4 foi composta por entrevistas semiestruturadas com pessoas trans. A coleta dos dados foi feita entre os dias 20 de maio de 2018 e 20 de maio de 2020. Por envolver coleta primária de dados, o estudo foi submetido à apreciação da Comissão Nacional de Ética em Pesquisa (CAAE – 85561717.0.0000.5149), sendo aprovado em 15 de maio de 2018. Todos os participantes tiveram acesso ao Termo de Consentimento Livre e Esclarecido e com ele concordaram.

Para este estudo em específico, a análise se vale de um conjunto de dados qualitativos formado por entrevistas semiestruturadas com pessoas trans maiores de 18 anos e residentes em dez municípios de Minas Gerais, que compõem a fase 4 da PMa. Foram entrevistados 15 indivíduos residentes em municípios-sede de sete das 13 macrorregiões de saúde do estado. As entrevistas foram realizadas de acordo com a disponibilidade de entrevistados e entrevistadores. 

Para a realização das entrevistas, foi utilizado um roteiro construído com base na literatura sobre os temas abordados no estudo (autopercepção de saúde, experiência de utilização dos serviços de saúde, experiências de não utilização dos serviços de saúde e saúde mental). 

A organização e investigação dos dados foi feita através do método análise de conteúdo (AC).^12^ Essa técnica de tratamento de dados tem o objetivo de interpretar as falas das pessoas entrevistadas por meio de uma descrição em consonância com as categorias de análise previamente definidas e/ou emergentes durante o processo. Além disso, permite uma leitura aprofundada e diversa dos dados coletados,^13^ que favorece a compreensão dos elementos da comunicação, como o “conteúdo manifesto ou latente e as significações explícitas ou ocultas”.^14^

Para potencializar a compreensão dos resultados das entrevistas, combinamos a estrutura da análise de conteúdo com as redes temáticas, uma ferramenta de análise qualitativa usada para organizar e representar temas emergentes de dados textuais, estruturando-os em três níveis: códigos básicos, temas organizadores e temas globais.^15^ Essa proposição analítica se caracteriza pela organização dos dados em uma rede de significados, levando em consideração seis passos principais: codificação do material; identificação dos temas; construção da rede temática; sua descrição e exploração; criação de uma estrutura resumo dos padrões e conexões dessas redes; e sua análise segundo os pressupostos teóricos da pesquisa.

Para se iniciar o processo de análise, todas as entrevistas foram transcritas literalmente e carregadas no *software* NVivo na versão 12 pro, a fim de agilizar, aumentar as possibilidades e garantir confiança à análise.^13^ As categorias para a codificação do material foram predefinidas com base na literatura levantada e, uma vez organizado o conteúdo das entrevistas em cada uma das categorias, foram utilizadas as ferramentas de visualização comparativa das categorizações para auxiliar na identificação dos temas e na construção da rede temática. 

## RESULTADOS

Conforme a Tabela 1, as 15 pessoas entrevistadas estão concentradas nos grupos etários mais jovens. Em relação às identidades de gênero, sete pessoas se classificaram como femininas, seis como masculinas e duas como agêneras/não binárias. No que se refere às orientações sexuais, oito pessoas reportaram ser heterossexuais, quatro bissexuais/pansexuais, uma lésbica, uma *gay* e uma demissexual.

**Tabela 1:** Composição das pessoas trans entrevistadas, Pesquisa Manas, Minas Gerais, 2018-2020

Identificação	Grupo etário	Identidade de gênero	Orientação sexual
T01	18-24	Mulher/feminino	Demissexual
T02	40+	Mulher/feminino	Heterossexual
T03	25-29	Mulher/feminino	Heterossexual
T04	25-29	Homem/masculino	Heterossexual
T05	25-29	Mulher/feminino	Heterossexual
T06	25-29	Homem/masculino	Bissexual/pansexual
T07	18-24	Homem/masculino	Heterossexual
T08	18-24	Agênera	Bissexual/pansexual
T09	40+	Mulher/feminino	Heterossexual
T10	40+	Mulher/feminino	Lésbica
T11	25-29	Homem/masculino	*Gay*
T12	18-24	Não binária	Bissexual/pansexual
T13	30-34	Homem/masculino	Heterossexual
T14	35-39	Homem/masculino	Heterossexual
T15	18-24	Travesti	Bissexual/pansexual

A análise das entrevistas resultou em uma rede temática composta por cinco temas (Figura 1). O primeiro, denominado *vivência*, aborda como as experiências vividas por essas pessoas foram moldadas e moldaram seus padrões de utilização e não utilização dos serviços de saúde, a percepção sobre seu estado de saúde, a resolutividade dos atendimentos e, principalmente, as violências físicas e simbólicas sofridas nesses espaços. Em seguida, apresentamos o segundo tema, com os relatos de como a *formação dos profissionais de saúde* (ou a falta de formação) afeta a predisposição das pessoas trans em procurar os serviços de saúde e o atendimento recebido. O terceiro tema, *redes de suporte*, aborda como as indicações de pares influencia as escolhas dos profissionais e de procedimentos, com ou sem supervisão médica e com ou sem condições financeiras para o custeio dos procedimentos desejados. O quarto tema aborda os desafios relacionados à *saúde mental*, em especial a obrigatoriedade do acompanhamento para o processo transexualizador e seus desdobramentos, bem como as barreiras para o acesso a esse tipo de serviço. Por fim, o quinto tema aborda a percepção das pessoas entrevistadas sobre o que seria um *atendimento ideal* para pessoas trans nos serviços de saúde. 

**Figura 1:** Rede temática, focando o acesso e a utilização dos serviços de saúde por pessoas trans, Pesquisa Manas, Minas Gerais, 2018-2020

As *vivências* da população trans nos serviços de saúde resultam de uma série de elementos sociais e contextuais, que moldam não só as suas percepções sobre “estar saudável”, mas essencialmente a relação entre os seus desejos e ideais de imagem e o que é ofertado nos serviços de saúde. Quando indagadas sobre a autoavaliação de saúde em comparação com outras pessoas trans da mesma faixa etária, o impacto das experiências comuns na vida das pessoas trans nos resultados de saúde fica mais evidente. As experiências de uso de hormônios sem acompanhamento, a aplicação de silicone industrial no corpo, o uso excessivo de drogas, a exposição aos riscos diversos vinculados às atividades de prostituição, a centralidade das mudanças corporais na construção da autoestima e os impactos do somatório de todas essas questões, ao longo da vida, são alguns dos pontos citados como justificativa para classificar a própria saúde (Quadro 1, painel a).

Mencionou-se também que, como muitas vezes a procura pelos serviços nem era realizada, práticas reconhecidamente desaconselháveis – como automedicação e outros procedimentos sem monitoramento de um profissional de saúde – são triviais entre eles e elas. No entanto, a autopercepção de saúde muitas vezes estava relacionada com a disponibilidade de atendimento. Para as pessoas que conseguem manter uma rotina de acompanhamento nos serviços de saúde combinada a práticas de autocuidado, parece haver uma ampliação do que se entende por ter uma boa saúde, vinculando-se ter uma boa saúde a uma utilização constante desses serviços (Quadro 1, painel b).

A maioria das demandas de saúde relatadas são referentes aos processos da transição – hormonização e cirurgias. Grande parte desses processos estão conectados ao desenvolvimento da autoestima com relação ao próprio corpo e das identidades trans. Contudo, mesmo com a criação dos serviços especializados para pessoas trans, eles ainda estão restritos aos grandes centros e enfrentam desafios para a provisão de serviços e insumos que atendam às demandas dessa população de forma regionalizada. Para conseguir atendimentos especializados, muitas pessoas se deslocam dos seus municípios e até estados. O processo transexualizador é restrito a poucos serviços, demorado e com longas filas de espera, que podem chegar a dez anos para os procedimentos cirúrgicos. É importante pontuar que a cirurgia não é uma necessidade de todas as pessoas trans. Em alguns relatos, é possível identificar experiências em que a cirurgia de redesignação sexual não é almejada e/ou não possui centralidade no processo de desenvolvimento das suas identidades (Quadro 1, painel c).

Com relação à experiência nos estabelecimentos, reconhece-se que os problemas enfrentados pela população geral, derivados de outras características sociais, como renda, raça/cor da pele e escolaridade, são interseccionados ao preconceito em relação às identidades trans. Para alguns dos entrevistados, o fato de ser trans torna pior a experiência de utilização dos serviços. No entanto, as identidades de gênero não foram em geral a razão pela qual o atendimento foi ruim, mas sim o fato de terem procurado um serviço de emergência e se depararem com equipamentos lotados e com pouca disponibilidade de profissionais para um atendimento adequado. A utilização dos serviços emergenciais, na maioria das vezes, pode ser fruto de um adiamento da busca por cuidados nos serviços de atenção primária à saúde (APS), motivada pelo receio dessas pessoas de serem mal atendidas por serem trans (Quadro 1, painel d).

A periodicidade em relação à utilização dos serviços de saúde também é heterogênea. Quando perguntadas sobre a última vez que foram a um serviço de saúde, algumas pessoas relataram que tiveram poucas experiências, há muito tempo, enquanto outras se disseram assíduas nos serviços de saúde e com uma experiência anterior recente. Por sua vez, a resolutividade dos atendimentos é percebida de forma positiva quando o acompanhamento leva em consideração tanto as demandas de hormonização como as condições gerais de saúde física e mental (Quadro 1, painel e).

A propensão de as pessoas trans buscarem atendimento engloba desde questões objetivas (por exemplo, desrespeito ao nome social nos serviços para aquelas que não retificaram os seus nomes no registro civil e falta de espaços reconhecidamente seguros e acolhedores para pessoas trans fora dos grandes centros) a questões sociais que, combinadas à qualidade do atendimento, geram uma sensação de que se trata de uma perda de tempo. Nesse mesmo sentido, as experiências negativas no sistema são permeadas por violações de direitos dentro e fora dos consultórios. Entre os relatos, foram citados a busca de cuidado especializado fora do município de residência, sem nenhum suporte do SUS, culminando em custos acima da renda disponível; desrespeito ao nome social, inclusive em equipamentos transespecíficos; condutas antiéticas e desrespeitosas dos profissionais durante o atendimento, como a recusa em prescrever hormônios e em realizar a mastectomia masculinizadora por não concordar com a retirada de uma mama saudável (Quadro 1, painel f).

Apesar da predominância de relatos negativos sobre o atendimento às pessoas trans, algumas experiências positivas também foram mencionadas, destacando-se a atuação do(da) profissional de saúde no acolhimento e cuidado das pessoas trans. A disponibilidade em buscar os melhores atendimentos possíveis para as demandas dos pacientes, a demonstração de domínio dos procedimentos e técnicas que seriam aplicadas, mas principalmente o tratamento humano e digno fizeram diferença, conforme o relato das pessoas trans. Para alguns, aspectos como ser uma pessoa conhecida e/ou pagar pelo atendimento podem ter auxiliado no processo (Quadro 1, painel g).

A *formação dos profissionais* é citada como um fator de extrema relevância para a experiência das pessoas trans nos serviços de saúde. Para muitas(os) entrevistadas(os), os profissionais que atuam hoje nos sistemas de saúde não possuem qualificação técnica adequada para lidar com as demandas da população LGBT+. Na maioria dos casos, os profissionais não possuem treinamento para acolher essa população da melhor forma. Os que possuem treinamento, em geral, são os únicos que atendem a esse tipo de demanda no serviço ou no município, e, quando propõem discussões sobre as demandas da população trans entre seus colegas, enfrentam resistência (Quadro 2, painel a). 

**Quadro 1:** Depoimentos selecionados sobre as vivências das pessoas trans nos serviços de saúde, Pesquisa Manas, Minas Gerais, 2018-2020

**a**	“Eu acho que [a minha saúde] está boa, eu não estou na rua tomando frio na pista [lugares de prostituição], sentindo frio à noite toda, estar ali na madrugada de roupa curta [...] eu já vi casos de mulheres trans na minha faixa [etária] que estava com a perna toda estropiada por causa de silicone industrial.” (*Mulher trans, branca, 40 anos ou mais)*
	“[os amigos trans] se hormonizam sozinhos, não têm acesso a ginecologista. O que precisa de medicamento consegue no mercado negro [sic], controla por conta própria a taxa de hormônio e isso é uma bomba que mexe com seu psicológico, mexe com emocional, mexe com tudo.” (*Homem trans, pardo, 30-34 anos*)
**b**	“Eu acho que apesar de tanto tempo tomando hormônio por conta própria, tem um ano mais ou menos que eu fiz um *check-up*, mas não deu ruim, poderia ter dado, ainda mais que eu tomava duas ampolas por semana e hoje eu tomo de 15 em 15 dias, é totalmente diferente, mas sim eu tenho uma boa saúde e tenho acesso.” (*Homem trans, preto, 35-39 anos*)
	“Meus exames, está tudo certo. Estou com o remédio certo, né?! Não a Perlutan, que é um anticoncepcional, faz um mal horrível, até para as mulheres cis.” (*Travesti, preta, 18-24 anos*)
**c**	“Não preciso de clínico geral porque eu não sinto nada, minha saúde é ótima. Preciso de acompanhamento endocrinológico e psicólogo, enquanto uma pessoa trans.” (*Travesti, preta, 18-24 anos*)
	“[...] no ambulatório basicamente é isso, só acompanhamento psicólogo, psiquiatra, porque o remédio mesmo a gente não tem liberado no SUS. Então, para as que não trabalham não tem condições de fazer uma transição pelo SUS.” (*Mulher trans, branca, 25-29 anos*)
	“Nosso único local que tem aqui mais próximo é BH que é o hospital de acompanhamento de transexualidade e tal, psicológico, hormonal, pra região aqui não tem nada, não tem nada.” (*Homem trans, pardo, 34-34 anos*)
**d**	“O tratamento foi bom, poderia ser melhor se tivesse médico [na UPA], estava em troca de plantão [...], mas eu não me senti excluída porque era para todos, eu estava ali igual a todo mundo.” (*Mulher trans, branca, 25-29 anos*)
	“[...] não tem [atendimento] adequado, não em função de ser trans não. É que o atendimento é ruim mesmo, muito superficial o atendimento em saúde no geral é ruim, mesmo as pessoas não trans. Médico nem olha na sua cara, tem muito disso, médico nem olha sabe, acho que é um problema generalizado e eu acho que as pessoas trans vão sofrer muito preconceito, mas eu provavelmente vou sofrer quando você mais está precisando.” (*Mulher trans, parda, 40 anos ou mais*)
**e**	“[...] eu tenho anos que não sei o que é um exame ginecológico. Um Papanicolau que é o que toda pessoa que tem um aparelho reprodutor feminino teria que fazer, não faço.” (*Homem trans, pardo, 34-34 anos*)
	“[...] eu usei o serviço de saúde ano passado, o PSF. Eu não vou sempre, mas uma ou duas vezes no ano.” (*Mulher trans, branca, 40 anos ou mais*)
	“Senti [diferença] totalmente. Eu fazia um ciclo [de hormonização], ela [médica] falou não vamos continuar com seu ciclo. Ela me passou um exame de sangue viu que não estava bom, ela mudou. E já deu muita mudança. De dezembro para agora é muita mudança.” (*Homem trans, branco, 18-24 anos*)
**f**	“Elas [amigas trans] têm receio, né? Não são bem tratadas. As que não procuram é porque não tem o nome ainda retificado. Então é mais receio, falta de respeito do profissional de saúde.” (*Mulher trans, branca, 40 anos ou mais*)
	“Vamos começar do recorte de classe. Sempre fui pobre, então acessar o serviço público de saúde para mim foi sempre muito moroso, sempre muito dificultoso. Então eu nunca tive essa cultura de ter uma amigdalite, ir ao posto de saúde, ou ter uma febre e ir na UPA. A gente corre no farmacêutico porque é mais rápido, mais barato, mais fácil, tem rotina, tem trabalho, não pode perder tempo com esse tipo de coisa.” (*Homem trans, pardo, 25-29 anos*)
	“[...] a gente tava lá e tal e eles chegaram e falaram assim “ah não, as pacientes da disforia de gênero”. Nos lugares você sempre tem um rótulo específico, você não tem a sua identidade.” (*Mulher trans, branca, 25-29 anos*)
**g**	“[...] ela [a atendente da policlínica] perguntou “porque ela não quer mostrar a identidade dela?” Ele [o médico ginecologista] disse que não precisa e me pegou e me levou. Ele é meu ginecologista até hoje, senão eu não tinha saído dali com hormônio, mas era uma época mais complicada 24 anos atrás.” (*Mulher trans, branca, 40 anos ou mais*)
	“[...] ele [o médico] não foi invasivo no sentido de tentar achar uma explicação para minha condição, ele me tratou como uma pessoa normal simplesmente, que é a pessoa que eu sou. [...] não me senti invadido, fui atendido enfim. O médico me pediu uma [quantidade grande] de exames, deu tudo ok, não deu alteração em nada, foi super tranquilo.” (*Homem trans, pardo, 25-29 anos*)

As dificuldades para encontrar profissionais adequados para lidar com questões específicas de saúde de pacientes trans decorre dessa falha na formação e falta de treinamento para o acolhimento dessa população. Dessa forma, para encontrar profissionais ou para se automedicar/realizar algum procedimento, esse grupo populacional, frequentemente, recorre às suas *redes de suporte*. Normalmente, essa indicação vem sempre de um(a) amigo(a) próximo(a) que já se consultou com esse profissional e se surpreendeu positivamente. Na ausência do cuidado, amigos(as) que vivenciam experiências semelhantes auxiliam na indicação de medicamentos sem prescrição médica ou na aplicação de silicone industrial (Quadro 2, painel b).

**Quadro 2:** Depoimentos selecionados sobre os fatores que influênciam a utilização dos serviços de saúde pelas pessoas trans, Pesquisa Manas, Minas Gerais, 2018-2020

a) Formação profissional	“[...] o médico que fez o atendimento comigo eu que tive que auxiliar. [Falar] qual era minha taxa que não podia abaixar daquilo senão ia menstruar, se passasse daquilo a testosterona virava progesterona e aí eu ia ter mais hormônio feminino. Eu tive que me sentar com o endócrino e informar isso. Então continuo fazendo sozinho sabe.” (Homem trans, pardo, 34-34 anos)
	“Porque, geralmente, a gente tem profissionais que não entendem, igual eu te falei. Não sabe qual nome dar. Já vi casos de meninas que o médico receitou, perguntando o que ela já tomava. E ficou por isso mesmo.” (Travesti, preta, 18-24 anos)
b) Redes de suporte	“[...] eu ouvi falar dele quando eu ia nas consultas com o psiquiatra, troquei ideia com algumas meninas que já tinham operado, dei uma pesquisada e ele me passou confiança.” (Mulher trans, branca, 25-29 anos)
	“[encontrei] através de grupos de BH de pessoas trans. Lá elas sempre jogam uma lista dos melhores médicos de BH e SP. Aí esse médico que eu procurei é um deles, que até não cobra [consulta]. Ele cobra de trans só a cirurgia, ele não cobra a ida lá, a consulta ele não cobra.” (Homem trans, preto, 35-39 anos)
	“[...] foi adequado, eu friso de novo, [mas] foi muito caro a consulta com ela. Essa médica é considerada a única em [do município de residência]. A que faz acompanhamento para mulheres, pessoas trans no geral. Mas assim ela é muito cara, pode ser muito inviável a consulta, o acompanhamento com ela.” (Mulher trans, raça não declarada, 18-24 anos)
	“[...] eu conseguindo a vaga no ambulatório, o hormônio é grátis, mas o problema é o transporte. Mesmo assim, ele sai no mesmo preço da cápsula, tipo uns 90 contos de transporte.” (Pessoa não binária, preta, 18-24 anos)
c) Saúde mental	“A parte da psicóloga lá é horrível. Você não fica dez minutos com ela na sala. Eu penso que é só burocracia. É oi, oi, tudo bem? tchau, assina aqui para mim.” (Homem trans, branco, 18-24 anos)
	“Eu já tive péssimas experiências com psicólogos, sabe?! Tipo de psicólogo de patologizar mesmo, sabe?! Não entender. E tratar a minha existência como uma questão universal, fora de todo o meu recorte social, recorte de gênero, recorte de identidade sexual, e tudo mais. O que foi péssimo!” (Travesti, preta, 18-24 anos)
	“[a psicóloga] me ajudou muito a suavizar algumas questões que eu trazia e algumas questões até meio estereotipadas do gênero feminino. Ela me ajudou a problematizar de uma maneira, não que fosse normativa ao contrário, mas de uma maneira sensível mesmo.” (Mulher trans, branca, 25-29 anos)
	“[...] quando você transiciona, não é só o seu exterior que vai mudar, isso tem impacto no lugar social. Você sai de um lugar e vai para outro. Antes eu era visto como mulher, então hoje em dia, por exemplo, eu saio na rua e não tenho medo de andar sozinho porque eu estou num lugar masculino. Todas essas coisas que te ultrapassam, te atravessam, você tem que entender. E entender isso sozinho às vezes não é muito fácil.” (Homem trans, pardo, 25-29 anos)
d) Atendimento ideal	“[...] que você tenha a atenção num tempo mínimo ali de convivência, sabe? [...] um atendimento que você consegue ter uma conversa e que não falta insumos para te atender.” (Pessoa agênera, branca, 18-24 anos)
	“Acho que o ideal é que entenda suas singularidades, mas sem tornar exótico. [...] que entenda que não é uma condição patológica, que as pessoas não vão estar confusas. [...] que considerem todo esse contexto social, histórico e político. Que de fato o problema não é da pessoa e o sofrimento não é só da pessoa ali. Ela não está sofrendo porque ela não ser aceita. Ela está sofrendo porque de fato tem todo um contexto que produz esse sofrimento nas pessoas. O sistema ideal tem que levar em consideração essas questões de saúde mental e como a sociedade de fato adoece as pessoas.” (Mulher trans, branca, 25-29 anos)
	“[...] não misturar princípios particulares com atendimento, porque no atendimento à saúde você encontra pessoas de vários tipos, de várias classes, de vários princípios.” (Homem trans, pardo, 34-34 anos)
	“Um atendimento que não te respeite com sua totalidade como ser humano.” (Homem trans, preto, 35-39 anos)

Apesar de a rede de suporte auxiliar na identificação dos profissionais e procedimentos para atender às demandas de saúde, nem sempre o custo é acessível. Em muitos casos, as pessoas buscam recursos financeiros com parentes, realizam a compra dos serviços de forma parcelada ou se esforçam para incluir esse custo no orçamento, aumentando o risco de empobrecimento. Além disso, mesmo quando os tratamentos são gratuitos, nem sempre são ofertados no município de residência, e os custos com transporte e hospedagem os tornam inviáveis em algumas situações (Quadro 2, painel b).

No que se refere à *saúde mental*, para alguns(algumas) entrevistados(as), a obrigatoriedade de acompanhamento psiquiátrico e psicológico para a realização das cirurgias de redesignação sexual pode servir como porta de entrada comum no sistema. Por outro lado, essa obrigatoriedade pode gerar, em alguns casos, atendimentos procedimentais, ou seja, protocolares, distanciando essa população dos serviços de saúde mental (Quadro 2, painel c).

Mesmo questionando grande parte dos processos que envolvem o acompanhamento psicológico compulsório, muitas entrevistadas reconhecem a importância e o papel desse acompanhamento no bem-estar de pessoas trans. Para algumas pessoas, o acompanhamento, quando bem-feito, auxilia não só na transição em si, mas também na forma de lidar com as demandas dos novos lugares sociais em que as pessoas trans passam a viver e com as mudanças contextuais que impactam suas vivências antes e após as cirurgias (Quadro 2, painel c).

Os relatos sobre o que seria um *atendimento ideal* para essa população apontam para um atendimento humanizado, que considere as especificidades das pessoas trans, sem se tentar enquadrá-las em uma explicação para sua condição ou tratamento para “solucionar o problema” (Quadro 2, painel d). 

Em termos gerais, o atendimento adequado é entendido como aquele em que se presta atenção genuína ao problema da pessoa, contando com recursos e tempo suficientes para uma interação significativa. Também é essencial que o atendimento seja imparcial e o paciente tratado com respeito e dignidade, considerando-se o histórico de vida e o estilo de vida das pessoas, e não apenas seus sintomas. Deve-se respeitar a identidade de cada pessoa, independentemente do que está nos documentos, e separar os princípios pessoais dos profissionais. Essas são atitudes fundamentais para um atendimento entendido como inclusivo e respeitoso.

## DISCUSSÃO

Este estudo teve como objetivo avaliar, sob a perspectiva de indivíduos trans, o acesso aos serviços de saúde. Nossos resultados vão ao encontro de outros estudos que apontam os principais aspectos que afetam a busca – ou não – por cuidado. Os principais fatores são a necessidade de estruturas adequadas para atendimento das demandas, incluindo o uso do nome social,^16-18^ o reconhecimento das demandas específicas com diagnóstico adequado,^19^ a falta de preparo dos profissionais,^17,18^ a centralidade das redes de suporte na busca por profissionais,^18^ o número elevado de automedicação e mudanças corporais sem acompanhamento médico,^20^ o contato – às vezes compulsório e inadequado – com os profissionais de saúde mental,^19^ o número reduzido e a concentração regional dos serviços que atendem às demandas do processo trasnsexualizador^2^ e a necessidade de implementação de uma visão integral do cuidado que não seja restrito ao processo transexualizador.^18^

Os resultados apontam para a necessidade de expandir estratégias de treinamento de profissionais de saúde em serviço e em formação no âmbito das políticas de saúde transespecíficas, sobretudo nos municípios de menor porte. A adoção dessas estratégias ampliaria o conhecimento e a resolução das demandas de saúde dessa população. A percepção de um cuidado de saúde resolutivo aumentaria a demanda pelos serviços, incluindo os da APS, o que propiciaria maior controle e prevenção das causas e fatores de risco de adoecimento dessa população. O acompanhamento adequado de pacientes trans também produziria efeitos do ponto de vista econômico, tanto da perspectiva individual quanto do sistema de saúde, na medida em que reduziria a necessidade de tratamentos mais complexos, caros e de longo prazo. 

As discussões apresentadas têm amplas implicações para as políticas de saúde trans, apontando para a necessidade de aprimoramento nos registros de informação para o uso do nome social e de inclusão da variável de identidade de gênero nos prontuários e cadastros, como o e-SUS. Essas medidas fortaleceriam a vigilância em saúde, proporcionando uma melhor compreensão do perfil epidemiológico da população trans. Também se destaca a necessidade de formação dos profissionais de saúde sobre identidades de gênero e sua relação com os determinantes sociais da saúde. Na assistência, é vital reconhecer e habilitar totalmente serviços transespecíficos do Estado e expandir o papel da APS na identificação e no acompanhamento da população trans. Isso garantiria uma compreensão precisa da demanda e da capacidade da APS na prescrição e acompanhamento da hormonioterapia para essa população nas unidades básicas de saúde. Essa indicação é defendida pela Sociedade Brasileira de Medicina de Família e Comunidade,^21^ que pontua que a hormonização pode ser realizada na APS com suporte de profissionais de níveis secundários e terciários quando necessário, reduzindo-se encaminhamentos para centros especializados e facilitando o acesso aos serviços. Essa abordagem é essencial, especialmente em Minas Gerais, dada a distribuição geográfica desigual dos serviços especializados no estado. Adicionalmente, é imperativo ampliar serviços de saúde mental, priorizando o respeito às identidades trans e abordando os impactos do estigma e do preconceito ao longo da vida.^22^

Esta pesquisa apresenta duas limitações importantes. O tamanho da amostra e a natureza não representativa destacam a necessidade de estudos futuros mais abrangentes. Outra limitação reside no contexto temporal, dado que as percepções e desafios podem evoluir ao longo do tempo. Pesquisas futuras devem abordar essas questões, explorando disparidades regionais e incorporando perspectivas dos profissionais de saúde para estratégias eficazes de melhoria do acesso de pessoas trans ao SUS. Apesar dessas limitações, os resultados encontrados proporcionam uma compreensão valiosa sobre o acesso à saúde para a população trans em Minas Gerais. A análise contribui para ampliar o debate, na medida em que considera um público pouco investigado, sobretudo entre residentes de cidades de pequeno e médio porte de diferentes regiões de Minas Gerais. Essas localidades, por estarem distantes de grandes centros, apresentam dificuldades de acesso que podem ser acentuadas devido à natureza dos cuidados de saúde necessários para atender às demandas desse grupo populacional.
